# Severe sudden sensorineural hearing loss related to risk of stroke and atherosclerosis

**DOI:** 10.1038/s41598-021-99731-w

**Published:** 2021-10-12

**Authors:** Nobuyoshi Tsuzuki, Koichiro Wasano, Naoki Oishi, Ko Hentona, Marie Shimanuki, Takanori Nishiyama, Yoshihiko Hiraga, Seiichi Shinden, Kaoru Ogawa

**Affiliations:** 1 Department of Otolaryngology, Head and Neck Surgery, Keio University School of Medicine, 35, Shinanomachi, Shinjuku, Tokyo, 160-8582 Japan; 2 Department of Otolaryngology, Hiratsuka City Hospital, 1-19-1 Minamihara, Hiratsuka-city, Kanagawa 254-0065 Japan; 3 National Institute of Sensory Organs, National Hospital Organization Tokyo Medical Center, 2-5-1 Higashigaoka, Meguro, Tokyo, 152-8902 Japan; 4 Department of Otolaryngology, National Hospital Organization Tokyo Medical Center, 2-5-1 Higashigaoka, Meguro, Tokyo, 152-8902 Japan; 5 Department of Otolaryngology, Saiseikai Utsunomiya Hospital, 911-1 Takebayashimachi, Utsunomiya-city, Tochigi 321-0974 Japan; 6 Department of Otolaryngology, Kawasaki Municipal Hospital, 12-1 Shinkawadori, Kawasaki, Kawasaki-city, Kanagawa 210-0013 Japan; 7 Department of Otolaryngology, Japanese Red Cross Shizuoka Hospital, 8-2 Outemachi, Aoi, Shizuoka-city, Shizuoka 420-0853 Japan

**Keywords:** Neurology, Risk factors

## Abstract

The cause of idiopathic sudden sensorineural hearing loss (idiopathic SSNHL)—diagnosed after excluding other causes of hearing loss, such as SSNHL associated with vestibular schwannoma (VS)—is unknown. The presumed pathogenesis of idiopathic SSNHL includes circulatory disorders (e.g., cochlear infarction). We tested the hypothesis that patients with SSNHL who are at high stroke risk will have a lower rate of VS compared to those with low stroke risk. The rationale is that the primary cause of SSNHL in patients with high stroke risk might be a circulatory disturbance. We conducted a retrospective study in six hospitals. Our sampling of SSNHL patients included those diagnosed with idiopathic SSNHL and VS-associated SSNHL. SSNHL patients who had a head MRI were stratified by severity of hearing loss and evaluated for differences in the detection rate of VS between the high-scoring CHADS_2_ (CHADS_2_-H-), an index of stroke risk, and low-scoring CHADS_2_ (CHADS_2_-L-) groups. We identified 916 patients who met the inclusion criteria. For severe hearing loss, the CHADS_2_-H group had a significantly lower rate of VS than the CHADS_2_-L group (OR 0 [95% CI 0.00–0.612]; *P* = 0.007). These results indirectly support the hypothesis that a primary cause of severe idiopathic SSNHL in those at high risk of stroke might be a circulatory disorder.

## Introduction

Idiopathic sudden sensorineural hearing loss (idiopathic SSNHL) is an abruptly appearing hearing loss that severely affects the quality of life of 11–77 per 100,000 people per year^1^. The hearing loss is often permanent and can be mild, total, or somewhere in between in severity. Idiopathic SSNHL is usually diagnosed after excluding other causes of SSNHL, such as vestibular schwannoma (VS). Although the exact cause of idiopathic SSNHL remains unknown, circulatory disorders, such as cochlear infarction, have been implicated in its pathogenesis^2^. This idea is consistent with findings from large cohort studies supporting the association of idiopathic SSNHL and stroke^3,4^. Moreover, comorbidities such as vertigo or dizziness and factors that increase risk of atherosclerosis, such as aging, diabetes mellitus, and history of cardiac disease, are reported to contribute to the severity of idiopathic SSNHL^5,6^. Despite these findings, there is still no definitive evidence that circulatory disturbances can cause idiopathic SSNHL.

SSNHL can be associated with a VS^7^. The prevalence of VS in patients with SSNHL is reported to be 0.8–3.7%^8^. Therefore, differentiation of retrocochlear pathology is necessary in the diagnosis of idiopathic SSNHL, and it is recommended that MRI or an auditory brainstem response test be conducted^9,10^.

Taken together, we formulated a hypothesis based on the following three steps. First, circulatory disturbances might be the primary cause of SSNHL in patients with high risk for stroke. Second, the cause of severe SSNHL in patients with high risk for stroke is even more likely to be circulatory disturbances, because a high risk of atherosclerosis contributes to the severity of SSNHL^5,6^. Third, therefore, severe SSNHL in patients at high risk of stroke are relatively less likely to have SSNHL caused by VS. In other words, we hypothesized that patients with severe SSNHL who are at high risk for stroke might have a lower rate of VS than those who are at low risk for stroke.

The CHADS_2_ score is one method for assessing stroke and atherosclerosis risk in patients with non-rheumatic atrial fibrillation^11^. CHADS_2_ is essentially a set of clinical prediction rules for estimating risk. CHADS_2_ stands for **C**ongestive heart failure, **H**ypertension, **A**ge, **D**iabetes mellitus, and **S**_**2**_ for prior stroke or transient ischemic attack. The CHADS_2_ score may be useful also as a predictor of cardiovascular/cerebrovascular events in patients with coronary artery disease lacking atrial fibrillation and as a prognosticator for patients with acute myocardial infarction^12,13^.

Thus, the present study tested this hypothesis by comparing the rate of VS in patients with SSNHL, who were stratified by CHADS_2_ score and severity of hearing loss. We then estimated the population of idiopathic SSNHL patients who are at risk for stroke and atherosclerosis by using CHADS_2_ scores and severity of hearing loss.

## Methods

We conducted a retrospective chart review of patients treated at six tertiary hospitals in Japan as part of the Keio Academical Otolaryngology Research Unit (KAORU) Project^7^. Patients diagnosed with SSNHL and who had undergone a head MRI in the departments of otolaryngology and head and neck surgery were eligible. Data were collected from the medical records of patients at the following hospitals and from the indicated time periods: Keio University Hospital between January 2012 and March 2020; National Hospital Organization Tokyo Medical Center between January 2012 and December 2019; Saiseikai Utsunomiya Hospital between January 2016 and November 2018; Japanese Red Cross Shizuoka Hospital between January 2014 and June 2019; Kawasaki Municipal Hospital between April 2016 and March 2020; and Hiratsuka City Hospital between January 2012 and December 2019.

Our sampling of SSNHL patients included those diagnosed with idiopathic SSNHL and VS-associated SSNHL. Idiopathic SSNHL was defined using the criteria of the Sudden Deafness Research Committee of the Ministry of Health, Labour and Welfare (MHLW), Japan (2015) (Table 1)^14^. VS-associated SSNHL was also defined according to these criteria, except for cases listed having an “unknown etiology” as main symptoms.

**Table 1:** Diagnostic Criteria for idiopathic Sudden Sensorineural Hearing Loss (idiopathic SSNHL).

**Main symptoms**
Sudden onset
Severe sensorineural hearing loss
Unknown etiology
**For reference**
Hearing loss (i.e., hearing loss of 30 dB or more over three consecutive frequencies within 72 h)
Exclude cases diagnosed as acute low-tone sensorineural hearing loss
Exclude functional hearing loss
Sudden onset of hearing loss; may progressively deteriorate over a few days
No repeated episodes of improvement or worsening of hearing loss
Unilateral hearing loss, but may be bilateral at the onset
May be accompanied by tinnitus around the time of onset of hearing loss
May be accompanied by vertigo, nausea, and/or vomiting around the time of onset of hearing loss, without recurrent episodes of vertigo
No cranial nerve symptoms other than from cranial nerve VIII
**Definite diagnosis:** All of the above main symptoms are present

*These criteria were established by the Research Committee of the Ministry of Health, Labour, and Welfare of Japan in 2015^14^.

Relevant data we collected were patient age, sex, affected ear (side), pure tone audiogram (PTA), presence of vertigo or dizziness symptoms, VS diagnosed by MRI; comorbidities (diabetes mellitus, hypertension, stroke/transient ischemic attack (TIA), congestive heart failure, vascular disease [e.g., myocardial infarction, angina pectoris, peripheral artery disease, aortic plaque], thromboembolism, hyperlipidemia, atrial fibrillation); and CHADS_2_ score. Scoring for CHADS_2_ was done by a physician; 1 point was assigned each for congestive heart failure, hypertension, age ≥ 75 years, or diabetes, and 2 points were assigned each for previous stroke and/or TIA^11^.

We excluded from analysis any patients diagnosed with bilateral simultaneous onset SSNHL. The first SSNHL post-onset PTA with complete values was used for analysis. The severity of PTA, which is the arithmetic mean of five hearing frequencies (250, 500, 1000, 2000, and 4000 Hz), was graded according to the criteria defined by the MHLW: Grade 1: PTA < 40 dB; Grade 2: 40 dB ≤ PTA < 60 dB; Grade 3: 60 dB ≤ PTA < 90 dB; and Grade 4: 90 dB ≤ PTA^14^.

For the first analysis, we classified SSNHL patients by presence/absence of VS and evaluated the two groups statistically according to various demographic and clinical characteristics. A receiver operating characteristic (ROC) curve was calculated with the CHADS_2_ scores for presence/absence of VS. The best cutoff value was determined using the Youden index^15^. With the ROC curve and Youden index analysis, we could identify the optimal cutoff value(s) that produced the best tradeoff between sensitivity and specificity.

Next, the patients were classified into a mild hearing-loss group (Grades 1 and 2) or a severe hearing-loss group (Grades 3 and 4). We evaluated the contribution of various risk factors for stroke and arteriosclerosis on the severity of SSNHL.

Finally, the patients were stratified by severity of hearing loss and evaluated for differences in the detection rate of VS between the CHADS_2_-H- and CHADS_2_-L-scoring groups.

The t-test and Fisher’s exact test were used for statistical analyses, as appropriate. P < 0.05 was defined as a statistically significant. We used EZR statistical software (Saitama Medical Center, Jichi Medical University, Saitama, Japan) for all analyses^16^. All methods of the present study were carried out in accordance with Strengthening the Reporting of Observational Studies in Epidemiology (STROBE) statement^17^.

All procedures were approved by the institutional review boards (IRBs) of the participating hospitals (IRB approval number): Keio University School of Medicine (20200033), National Hospital Organization Tokyo Medical Center (R20-046), Saiseikai Utsunomiya Hospital (2020-19), Japanese Red Cross Shizuoka Hospital (2020-15), Kawasaki Municipal Hospital (2020-9), and Hiratsuka City Hospital (02-003). The requirement of written informed consent was waived by the IRBs of Keio University School of Medicine, National Hospital Organization Tokyo Medical Center, Saiseikai Utsunomiya Hospital, Japanese Red Cross Shizuoka Hospital, Kawasaki Municipal Hospital, and Hiratsuka City Hospital, because of the retrospective design.

## Results

We identified 916 SSNHL patients (426 men [46.5%], 490 women [53.5%]) who met the inclusion criteria. Mean age ± standard deviation (SD) at the time of the initial examination was 59.4 ± 15.4 years.

Table 2 shows the demographic and clinical characteristics of included patients with SSNHL, stratified by presence/absence of VS. SSNHL patients with VS and those without VS showed no significant differences across any of their demographic and clinical characteristics. Using ROC curve analysis, we determined that the optimal cutoff values for the CHADS_2_ score were at two points (CHADS_2_-H, CHADS_2_-L) on the specificity-sensitivity curve. The area under the curve (AUC) was 0.551 (95% confidence interval (CI), 0.487–0.616). This categorization of CHADS_2_ scores into low and high groups by cutoff value has been reported in several reports^11,13^. We found no significant difference in the rate of CHADS_2_-H and CHADS_2_-L between SSNHL patients with VS and those without VS (p = 0.06) (odds ratio (OR), 0.38 [95% CI 0.099–1.066]).

**Table 2:** Demographic and clinical characteristics of included SSNHL patients with and without vestibular schwannoma.

Characteristic	VS- (n = 863)	VS + (n = 53)	OR	95% CI	p value*	Statistical test used
Mean age (SD), years	59.6 (15.3)	56.5 (16.6)			0.17	t-test
**Sex**						
Male	404 (46.8%)	22 (41.5%)	1	(Reference)	0.48	Fisher's exact test
Female	459 (53.2%)	31 (58.5%)	1.24	0.68–2.29		
**Affected ear**						
Left	459 (53.2%)	31 (58.5%)	1	(Reference)	0.48	Fisher's exact test
Right	404 (46.8%)	22 (41.5%)	0.81	0.44–1.46		
**Initial grade of hearing loss**^**†**^						
Grade 1	171 (19.8%)	10 (18.9%)			0.75	Fisher's exact test
Grade 2	262 (30.4%)	20 (37.7%)				
Grade 3	310 (35.9%)	17 (32.1%)				
Grade 4	120 (13.9%)	6 (11.3%)				
**Symptom**						
Vertigo/dizziness	251 (29.1%)	19 (35.8%)	1.36	0.72–2.51	0.35	Fisher's exact test
**Comorbidities**						
Diabetes mellitus	140 (16.2%)	5 (9.4%)	0.54	0.16–1.38	0.24	Fisher's exact test
Hypertension	243 (28.2%)	12 (22.6%)	0.75	0.35–1.48	0.43	Fisher's exact test
Stroke/TIA	30 (3.5%)	0 (0%)	0	0–2.13	0.41	Fisher's exact test
Congestive heart failure	21 (2.4%)	0 (0%)	0	0–3.15	0.63	Fisher's exact test
Vascular disease	67 (7.8%)	2 (3.8%)	0.47	0.05–1.84	0.42	Fisher's exact test
Thromboembolism	9 (1.0%)	1 (1.9%)	1.82	0.04–13.60	0.45	Fisher's exact test
Hyperlipidemia	192 (22.2%)	10 (18.9%)	0.81	0.36–1.68	0.73	Fisher's exact test
Atrial fibrillation	18 (2.1%)	1 (1.9%)	0.90	0.02–5.95	1	Fisher's exact test
**CHADS**_**2**_** score**^**§**^						
0	478 (55.4%)	33 (62.3%)			0.72	Fisher's exact test
1	233 (27.0%)	16 (30.2%)				
2	105 (12.2%)	3 (5.7%)				
3	32 (3.7%)	1 (1.9%)				
4	10 (1.2%)	0 (0%)				
5	4 (0.5%)	0 (0%)				
6	1 (0.1%)	0 (0%)				
0 or 1 (CHADS_2_-L)	711(82.4%)	49(92.5%)	1	(Reference)	0.06	Fisher's exact test
2 or more (CHADS_2_-H)	152(17.6%)	4(7.5%)	0.38	0.10–1.07		

*SD* standard deviation, *TIA* transient ischemic attack, *VS* vestibular schwannoma, *VS* + confirmed vestibular schwannoma by MRI, *VS − *no evidence of vestibular schwannoma, *OR* odd ratio, *95% CI* 95% confidence interval, *CHADS*_*2*_*-L* low-scoring CHADS_2_, *CHADS*_*2*_*-H* high-scoring CHADS_2_.

*Significance level: p < 0.05.

^†^Graded according to the criteria defined by the Sudden Deafness Research Committee of the Ministry of Health, Labour and Welfare (MHLW), Japan: Grade 1: PTA < 40 dB; Grade 2: 40 dB ≤ PTA < 60 dB; Grade 3: 60 dB ≤ PTA < 90 dB; and Grade 4: 90 dB ≤ PTA^14^.

^§^A high CHADS_2_ score corresponds to a greater risk of stroke and a low score, a lower risk^11^.

Table 3 shows summaries of the analysis of patient characteristics associated with severity of SSNHL. We found no significant difference in the rate of VS between the Grade 1–2 severity group and Grade 3–4 severity group (p = 0.40) (OR 0.77 [95% CI 0.421–1.400]). Patients in the Grade 3–4 group were significantly older; had more vertigo or dizziness symptoms; were more likely to have diabetes mellitus, hypertension, congestive heart failure, vascular disease, and/or atrial fibrillation; and had higher CHADS_2_ scores compared with patients in the Grade 1–2 group.

**Table 3:** Characteristics and Comorbidities of Patients with SSNHL Stratified by Hearing Loss Severity Grade 1–2 and Grade 3–4.

Characteristic	Grade 1–2^†^ (n = 463)	Grade 3–4^†^ (n = 453)	OR	95% CI	p value^‡^	Statistical test used
**VS**					0.40	Fisher's exact test
Absent	433 (93.5%)	430 (94.9%)	1	(Reference)		
Present	30 (6.5%)	23 (5.1%)	0.77	0.42–1.40		
Mean age (SD), years	58.1 (14.7)	60.6 (16.0)			0.01*	t-test
**Sex**					0.60	Fisher's exact test
Male	211 (45.6%)	215 (47.5%)	1	(Reference)		
Female	252 (54.4%)	238 (52.5%)	0.93	0.71–1.21		
**Affected ear**					< 0.01**	Fisher's exact test
Left	269 (58.1%)	221 (48.8%)	1	(Reference)		
Right	194 (41.9%)	232 (51.2%)	1.45	1.11–1.91		
**Symptom**						
Vertigo/dizziness	104 (22.5%)	166 (36.6%)	2.00	1.48–2.70	< 0.01**	Fisher's exact test
**Comorbidities**						
Diabetes mellitus	58 (12.5%)	87 (19.2%)	1.66	1.14–2.43	< 0.01**	Fisher's exact test
Hypertension	114 (24.6%)	141 (31.1%)	1.38	1.02–1.87	0.03*	Fisher's exact test
Stroke/TIA	11 (2.4%)	19 (4.2%)	1.80	0.80–4.23	0.14	Fisher's exact test
Congestive heart failure	2 (0.4%)	19 (4.2%)	10.10	2.41–89.93	< 0.01**	Fisher's exact test
Vascular disease	15 (3.2%)	54 (11.9%)	4.04	2.20–7.83	< 0.01**	Fisher's exact test
Thromboembolism	3 (0.6%)	7 (1.5%)	2.40	0.54–14.50	0.22	Fisher's exact test
Hyperlipidemia	93 (20.1%)	109 (24.1%)	1.26	0.91–1.75	0.15	Fisher's exact test
Atrial fibrillation	4 (0.9%)	15 (3.3%)	3.93	1.24–16.36	0.01*	Fisher's exact test
**CHADS**_**2**_** score**^**§**^					< 0.01**	Fisher's exact test
0 or 1 (CHADS_2_-L)	404 (87.3%)	356 (78.6%)	1	(Reference)		
2 or more (CHADS_2_-H)	59 (12.7%)	97 (21.4%)	1.86	1.29–2.71		

*SD* standard deviation, *TIA* transient ischemic attack, *VS* vestibular schwannoma, *VS* + confirmed vestibular schwannoma by MRI, *VS − *no evidence of vestibular schwannoma, *OR* odd ratio, *95% CI* 95% confidence interval, *CHADS*_*2*_*-L* low-scoring CHADS_2_, *CHADS*_*2*_*-H* high-scoring CHADS_2_.

^†^Graded according to the criteria defined by the Sudden Deafness Research Committee of the Ministry of Health, Labour and Welfare (MHLW), Japan: Grade 1: PTA < 40 dB; Grade 2: 40 dB ≤ PTA < 60 dB; Grade 3: 60 dB ≤ PTA < 90 dB; and Grade 4: 90 dB ≤ PTA^14^.

^‡^Significance level: *p < 0.05, ** p < 0.01.

^§^A high CHADS_2_ score (2 or more) corresponds to a greater risk of stroke and a low score (0 or 1), a lower risk^11^.

**Figure 1:**
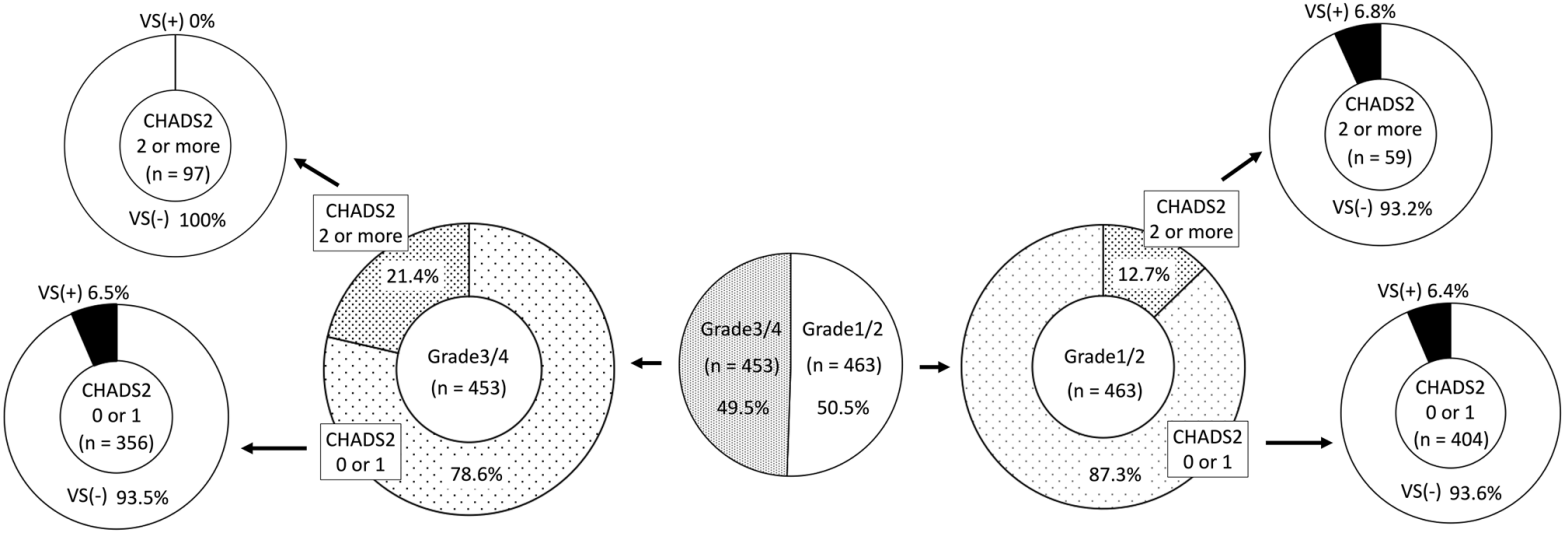
Schematic diagram showing the distribution of SSNHL patients stratified by hearing loss grade, CHADS_2_ score, and presence or absence of vestibular schwannoma. No vestibular schwannoma (VS) was detected in Grade 3–4 patients with a CHADS_2_ score of 2 or more.

Figure 1 shows the prevalence of VS in SSNHL patients, stratified by the severity of hearing loss and CHADS_2_ score. The CHADS_2_-H group had a lower prevalence rate of VS than the CHADS_2_-L group, although this difference was not statistically significant (p = 0.06) (OR, 0.38 [95% CI 0.099–1.066]). However, in the Grade 3–4 group, the CHADS_2_-H group had a significantly lower prevalence rate of VS than the CHADS_2_-L group (p = 0.007) (OR, 0 [95% CI 0.00–0.612]). In the Grade 1–2 group, CHADS_2_-H and CHADS_2_-L participants were statistically indistinguishable regarding rate of VS (p = 1) (OR 1.06 [95% CI 0.258–3.216]).

## Discussion

Idiopathic SSNHL is an abruptly appearing and disabling inner ear disorder that presents mostly unilaterally. The pathogenesis of idiopathic SSNHL has not been identified. VS, viral infection (e.g. mumps virus), intralabyrinthine hemorrhage and rupture of the labyrinthine window have been reported as causes of SSNHL^7,18–20^. Previous studies have suggested that risk factors for cerebrovascular and cardiovascular disease can also be considered risk factors for idiopathic SSNHL^21,22^. Although previous studies have provided evidence for the hypothesis that idiopathic SSNHL is caused by a circulatory disturbance^2–4^, like cochlear infarction, no definitive conclusions have been reached. In the present study, we evaluated associations of stroke and atherosclerosis risk using patients’ CHADS_2_ scores and tested whether the rate of VS in SSNHL patients differed systematically according to CHADS_2_ score. Our aim was to determine the population of idiopathic SSNHL patients who had higher stroke and atherosclerosis risk by excluding from analysis SSNHL patients with VS.

We had two main findings. First, we found an association between CHADS_2_ scores and severity of hearing loss in SSNHL patients. CHADS_2_ scores of SSNHL patients with Grade 1–2 hearing loss were significantly different from those with Grade 3–4 hearing loss. Other significant differences were found in proportion with vertigo/dizziness; age; and comorbidities (diabetes mellitus, hypertension, congestive heart failure, vascular disease, and atrial fibrillation). These results suggest that patients with Grade 3–4 hearing loss severity were more likely to have higher risk factors for stroke/atherosclerosis, which is consistent with previous reports^5,6^. Furthermore, when stratifying SSNHL patients by hearing-loss grade and CHADS_2_ score, participants in the CHADS_2_-H group with Grade 3–4 severity were significantly less likely to have a VS than those Grade 3–4 severity participants in the CHADS_2_-L group. Indeed, no VS was detected in Grade 3–4 patients with high risk factors for stroke/atherosclerosis. This may be an important result to more clearly define the population of idiopathic SSNHL patients associated with stroke and atherosclerosis risk.

Second, the rate of VS in SSNHL patients was 5.8%, which was slightly higher than previously reported^8^. We speculate that this is because the criteria defined by the MHLW in Japan are strictly applied in the diagnosis of idiopathic SSNHL. In the present study, patients with acute low-frequency sensorineural hearing loss (ALHL) limited to 125 to 500 Hz were excluded. A past report indicates that the rate of VS in ALHL cases is lower than the rate of VS in all SSNHL cases^23,24^. In other words, the exclusion of SSNHL patients who did not meet this criterion, such as ALHL cases limited to 125 to 500 Hz, may have resulted in a slightly higher VS rate. Some reports suggest that all cases of SSNHL should be screened for VS by MRI^25^. However, our present results suggest that MRI screening for SSNHL of patients with a CHADS_2_ score of ≥ 2 accompanied by Grade 3–4 severity might not have higher priority. This population accounted for 10.6% of the SSNHL patients (97/916 cases).

The present study has three possible limitations. First, the study is retrospective, and therefore may have selection bias and information bias. Second, the factors and risks of VS are still largely unknown and could not be adjusted for in the analysis. Third, idiopathic SSNHL itself is a disorder whose pathogenesis is still unknown.

The present study also had at least two strengths. First, it was a multicenter study with a relatively large number of patients. Second, for the first time, our analysis allowed an indirect estimate of the presumed population of SSNHL patients associated with stroke and atherosclerosis risk according to severity of hearing loss and CHADS_2_ score.

In conclusion, patients with severe hearing loss (Grade 3–4) and high CHADS_2_ scores had a significantly lower rate of VS than SSNHL patients with low CHADS_2_ scores. By process of elimination, this suggests that the cause of severe idiopathic SSNHL in individuals at high risk of stroke and atherosclerosis might be a circulatory disorder. Thus, when determining whether the severe hearing loss in a patient is due to idiopathic SSNHL, clinicians should calculate the patient’s CHADS_2_ score and then decide whether the patient should undergo an MRI to rule out VS. If the CHADS_2_ score is high, the patient should undergo whole brain MRI and/or MR angiography. We hypothesize that the cause of severe SSNHL in patients with a high risk for stroke/atherosclerosis is a circulatory disturbance, which we will evaluate in the near future.

## Data Availability

The data analysed during this study are available from the corresponding author upon reasonable request.
